# Stimuli-responsive polypeptide nanogels for trypsin inhibition

**DOI:** 10.3762/bjnano.13.45

**Published:** 2022-06-22

**Authors:** Petr Šálek, Jana Dvořáková, Sviatoslav Hladysh, Diana Oleshchuk, Ewa Pavlova, Jan Kučka, Vladimír Proks

**Affiliations:** 1 Institute of Macromolecular Chemistry, Academy of Sciences of the Czech Republic, Heyrovského nám. 2, 162 06 Prague 6, Czech Republic; 2 Department of Physical and Macromolecular Chemistry, Faculty of Science, Charles University, Hlavova 8, 128 40 Prague 2, Czech Republic

**Keywords:** α_1_-antitrypsin, inflammatory mediator, nanogel, polypeptide, trypsin

## Abstract

A new type of hydrophilic, biocompatible, and biodegradable polypeptide nanogel depots loaded with the natural serine protease inhibitor α1-antitrypsin (AAT) was applied for the inhibition of the inflammatory mediator trypsin. Two types of nanogels were prepared from linear synthetic polypeptides based on biocompatible and biodegradable poly[*N*^5^-(2-hydroxyethyl)-ʟ-glutamine-*ran*-*N*^5^-propargyl-ʟ-glutamine-*ran*-*N*^5^-(6-aminohexyl)-ʟ-glutamine]-*ran*-*N*^5^-[2-(4-hydroxyphenyl)ethyl)-ʟ-glutamine] (PHEG-Tyr) or biocompatible *N*^α^-ʟ-lysine-grafted α,β-poly[(2-propyne)-ᴅ,ʟ-aspartamide-*ran*-(2-hydroxyethyl)-ᴅʟ-aspartamide-*ran*-(2-(4-hydroxyphenyl)ethyl)-ᴅʟ-aspartamide] (*N*^α^-Lys-NG). Both nanogels were prepared by HRP/H_2_O_2_-mediated crosslinking in inverse miniemulsions with pH and temperature-stimuli responsive behavior confirmed by dynamic light scattering and zeta potential measurements. The loading capacity of PHEG-Tyr and *N*^α^-Lys-NG nanogels and their release profiles were first optimized with bovine serum albumin. The nanogels were then used for loading and release of AAT. PHEG-Tyr and *N*^α^-Lys-NG nanogels showed different loading capacities for AAT with the maximum (20%) achieved with *N*^α^-Lys-NG nanogel. In both cases, the nanogel depots demonstrated a burst release of AAT during the first 6 h, which could be favorable for quick inhibition of trypsin. A consequent pilot in vitro inhibition study revealed that both PHEG-Tyr and *N*^α^-Lys-NG nanogels loaded with AAT successfully inhibited the enzymatic activity of trypsin. Furthermore, the inhibitory efficiency of the AAT-loaded nanogels was higher than that of only AAT. Interestingly, also non-loaded PHEG-Tyr and *N*^α^-Lys-NG nanogels were shown to effectively inhibit trypsin because they contain suitable amino acids in their structures that effectively block the active site of trypsin.

## Introduction

Despite significant and progressive advances in medicine, the global incidence of pancreatitis shows that this disease remains one of the most severe health problems worldwide [1]. Pancreatitis is an inflammatory disease of the pancreas, categorized as either acute pancreatitis, which occurs suddenly as a short-term illness, or chronic pancreatitis, which is a long-lasting condition. Acute pancreatitis may lead to chronic pancreatitis with a high risk of consecutive development of cancer [2–3]. The pancreas has exocrine and endocrine functions of which the exocrine function serves to secrete digestive enzymes, such as trypsin, into the intestine. Trypsin belongs to a group of serine proteases and is excreted by the pancreas in an inactive form known as trypsinogen with subsequent activation in the duodenum. However, premature activation of trypsinogen in pancreatic acinar cells causes an inflammatory process in which trypsin induces proteolysis, pancreatic injury, and the onset of pancreatitis [4–5].

Various organisms naturally produce inhibitors of serine proteases to regulate the inappropriate proteolytic activity that may lead to undesirable inflammatory processes [6]. Beneficial biological activity of serine protease inhibitors has been successfully exploited for therapeutic purposes or antinutritional properties [7–8]. Different types of small proteins or polypeptides represent a class of bioactive compounds identified as serine protease inhibitors. Aprotinin, a Kunitz-type serine protease inhibitor, is well known for its inhibitory activity regarding trypsin [9]. Other examples are ulinastatin, a glycoprotein found in urine, plasma, and all organs, and gabexate, a synthetic serine protease inhibitor that inhibits secretion and activity of trypsin. Both are successfully used for the treatment of acute pancreatitis [10]. The pancreas itself secretes the serine protease inhibitor Kazal type 1 (SPINK1) to inhibit trypsinogen autoactivation and proteolytic activity of trypsin. The loss in SPINK1 secretion and function increases the risk of pancreatitis development [4]. As a defense mechanism against serine proteases and other inflammatory mediators, organisms secrete many other serine proteases inhibitors, such as α_1_-antitrypsin (AAT), α_2_-macroglobulin, α_1_-antichymotrypsin, and eglin C [11]. However, direct intravenous or oral administration of the majority of these natural serine protease inhibitors is ineffective due to their low plasma and peritoneal levels, very short in vivo half-lives, chemical instability, and high susceptibility to degradation [6].

Delivery systems represented by various polymer particles provide necessary and effective protection of bioactive cargo against degradation and elimination as well as for prolonged circulation time. Nanogels, soft hydrogel nanoparticles, have been proven efficient carriers of proteins or peptides preserving the biological activity of their payload [12–14]. For instance, Ozawa et al. introduced a nanogel from highly branched cyclic dextrin derivatives that trapped fluorescein isothiocyanate-labeled insulin, which was continuously released over 12 h [15]. Hirakura et al. fabricated a cholesteryl group-bearing pullulan nanogel serving as a reservoir of three different proteins, glucagon-like peptide 1, insulin, and erythropoietin incorporated in hyaluronan hydrogel [16]. Morimoto et al. prepared an acid-labile cholesteryl-modified pullulan nanogel that formed a complex with loaded bovine serum albumin and released it under acidic conditions [17]. Landfester et al. used a bio-orthogonal reaction to fabricate a dextran nanogel with pH-responsive hydrazine linkages allowing for a controlled release of loaded FITC-albumin [18]. Regarding the inhibition of serine proteases and inflammation, AAT, the most abundant inhibitor of serine proteases in human plasma, regulates the proteolytic activity of secreted proteases and is involved in the acute anti-inflammatory response against inflammatory mediators [19]. Interestingly, it was revealed that subjects with AAT deficiency suffer from increased activation of neutrophils, levels of cytokines, and inflammation, and that AAT administration can slow down a decrease in insulin production during diabetes [20–21]. A few poly (ᴅ,ʟ-lactide-*co*-glycolide) (PLGA) nanoparticles loaded with AAT were successfully manufactured and AAT release profiles from the nanoparticles were investigated [21–22].

In our previous studies, we investigated and described in detail the process of nanogelation from *N*^α^-ʟ-lysine-grafted α,β-poly[(2-propyne)-ᴅ,ʟ-aspartamide-*ran*-(2-hydroxyethyl)-ᴅʟ-aspartamide-*ran*-(2-(4-hydroxyphenyl)ethyl)-ᴅʟ-aspartamide] (*N*^α^-Lys-NG) and poly[*N*^5^-(2-hydroxyethyl)-ʟ-glutamine-*ran*-*N*^5^-propargyl-ʟ-glutamine-*ran*-*N*^5^-(6-aminohexyl)-ʟ-glutamine]-*ran*-*N*^5^-[2-(4-hydroxyphenyl)ethyl)-ʟ-glutamine] (PHEG-Tyr) polypeptide precursors by HRP/H_2_O_2_-mediated crosslinking in inverse miniemulsion including the synthetics for these polymer precursors [23–25]. Our effort resulted in the development of new types of polypeptide nanogels with potential for biological applications [24–25]. In this work, we have designed two types of nanogels from biocompatible and biodegradable PHEG-Tyr and biocompatible and non-biodegradable *N*^α^-Lys-NG polypeptide precursors by HRP/H_2_O_2_-mediated crosslinking in inverse miniemulsion for the delivery of AAT. Both prepared polypeptide nanogels demonstrate stimuli-responsive behavior under different pH values and temperatures. After optimizing the loading and release from PHEG-Tyr and *N*^α^-Lys-NG with bovine serum albumin (BSA), loading and release of AAT were investigated. The results show that AAT has a higher affinity to *N*^α^-Lys-NG and both nanogel depots demonstrate burst release over 6 h followed by continuous release over ca. 160 h. Finally, the PHEG-Tyr and *N*^α^-Lys-NG nanogels loaded with AAT were successfully applied for pilot in vitro inhibition studies of trypsin activity.

## Results and Discussion

### Preparation of the nanogels

**Figure 1:**
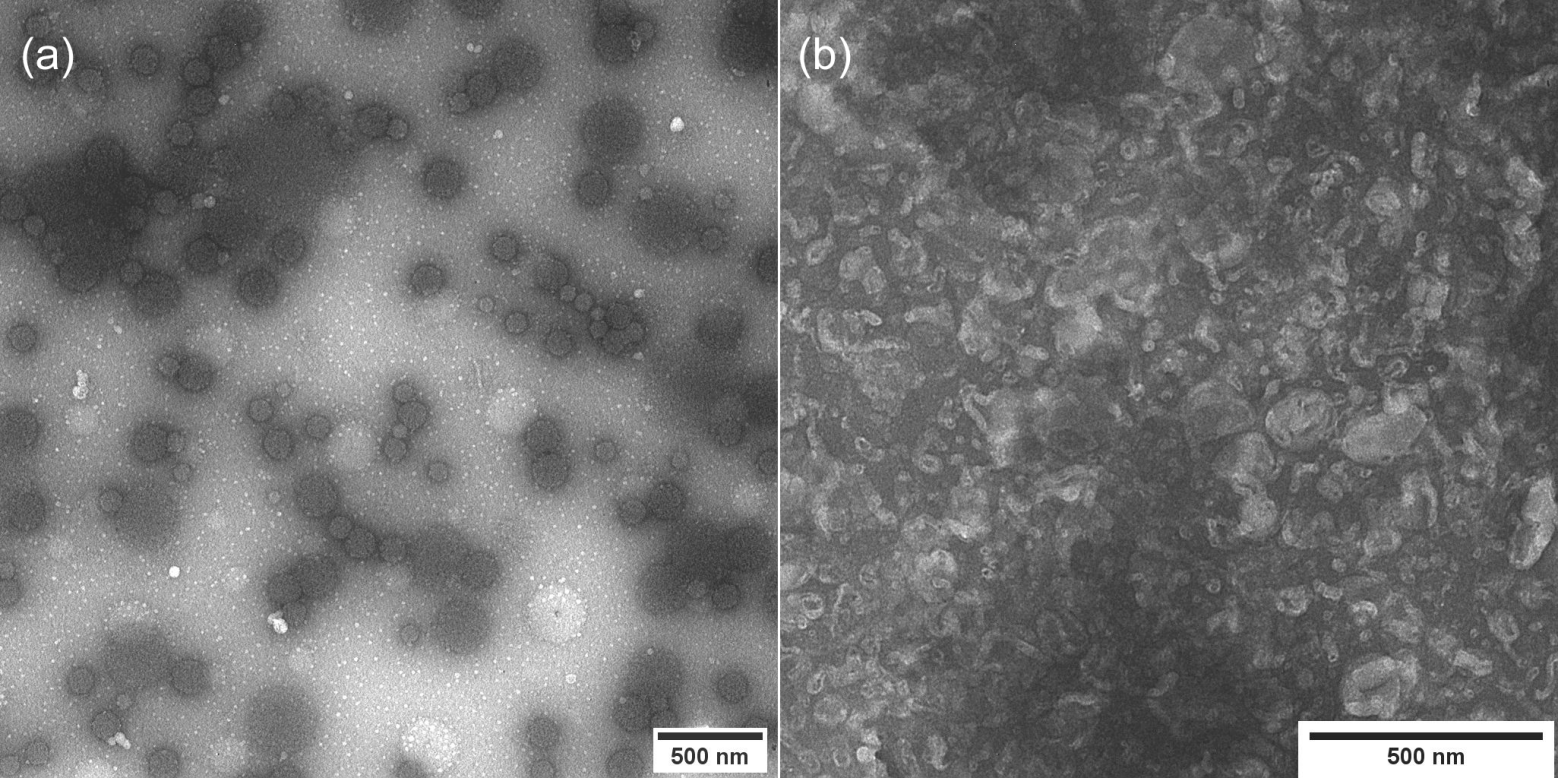
TEM images of PHEG-Tyr (a) and *N*^α^-Lys-NG (b) nanogels prepared by HRP/H_2_O_2_-mediated crosslinking in inverse miniemulsion.

The synthetic polypeptides polyglutamine PHEG-Tyr and zwitterionic polyaspartamide *N*^α^-Lys-NG, which were prepared and characterized according to earlier published procedures [23–25], were nanogelated in inverse miniemulsion via HRP/H_2_O_2_ crosslinking to obtain chemically dityramine-crosslinked nanogels [23]. Our previous studies examined the effect of different surfactants on the final properties of PHEG-Tyr nanogel, showing that in the presence of TWEEN 85 surfactant a spherical nanogel with low yield was obtained. In contrast, in the presence of SPAN 80 surfactant, the final nanogel was more irregular in shape while the yield of nanogelation was increased. The in vitro and in vivo tests proved biocompatibility and biodegradability of PHEG-Tyr nanogel and biocompatibility of *N*^α^-Lys-NG nanogel [23–25]. In this study, we attempt to obtain improved shape and morphology of PHEG-Tyr nanogel by combining surfactant SPAN 80 with surfactant TWEEN 85 based on a previous study presenting the stabilization effect of TWEEN 85 on HRP/H_2_O_2_-mediated nanogelation in inverse miniemulsion [24]. The addition of TWEEN 85 is shown to better support the stabilization effect of SPAN 80. The prepared PHEG-Tyr nanogel is spherical in shape and more regular (Figure 1a) than the PHEG-Tyr nanogel prepared only in the presence of SPAN 80 [24]. TEM microscopy analysis has revealed a slight narrowing of the particle size distribution with *Đ* = 1.43. PHEG-Tyr nanogel is composed of two families of compact hydrogel spheres with *D*_n_ = 111 and 19 nm, and *D*_w_ = 159 and 24 nm, respectively. Biocompatible zwitterionic *N*^α^-Lys-NG was synthesized according to our earlier study [25] and TEM image analysis of *N*^α^-Lys-NG demonstrated that the nanogel is more irregular in shape and collapses in the dry state (Figure 1b). This can be the result of the softer structure of *N*^α^-Lys-NG nanogel with *D*_n_ values in the range of 50 to 180 nm.

### Stimuli-responsive behavior of the nanogels

**Figure 2:**
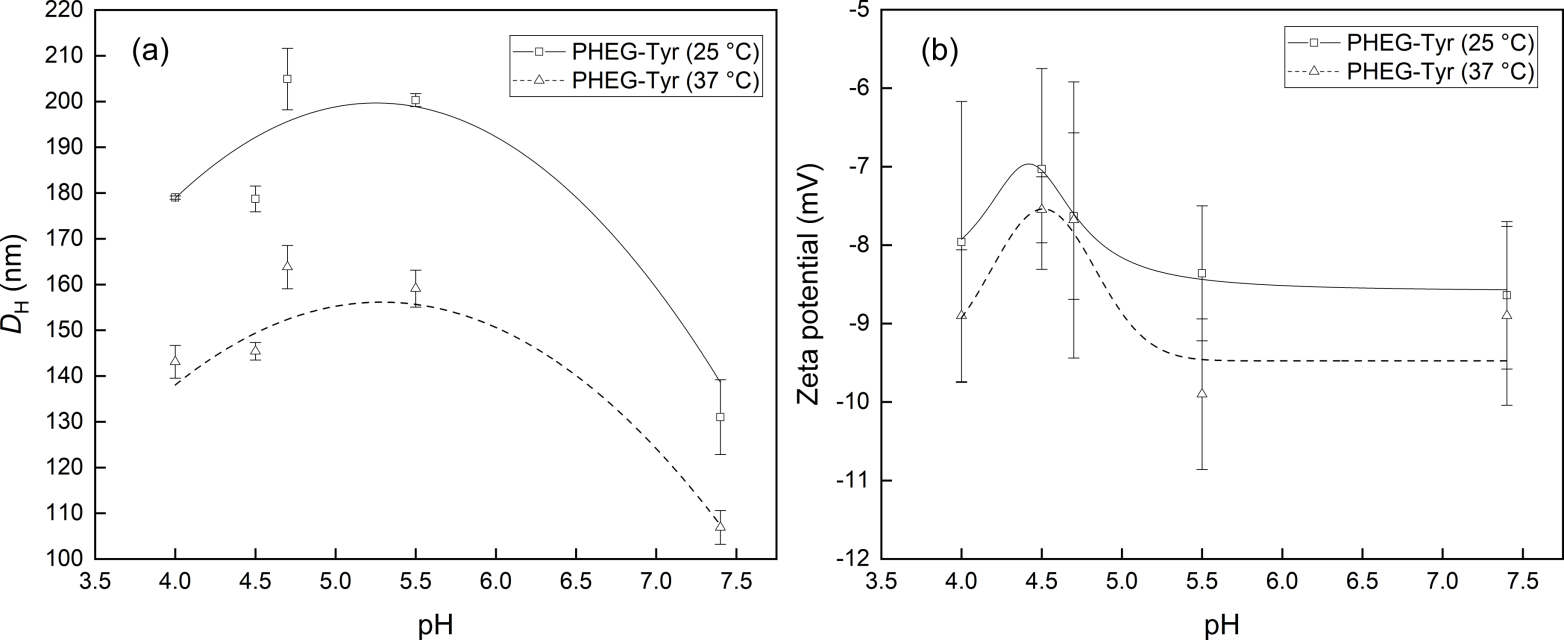
Dependence on the pH value of *D*_H_ (a) and zeta potential (b) of PHEG-Tyr nanogel at 25 (squares) and 37 °C (triangles). The lines are to guide the eye.

As the formed polypeptides are polyelectrolytes, their stimuli-responsive behavior was studied [26]. Here, biocompatible and biodegradable PHEG-Tyr and biocompatible *N*^α^-Lys-NG were incubated at different pH values and temperatures to study their physicochemical properties represented by *D*_H_ and zeta potential. Figure 2a depicts the change in *D*_H_ of PHEG-Tyr nanogel as a response to pH variation from 4 to 4.7 at 25 °C, showing a continuous increase of *D*_H_ from 179 to 205 nm as a result of the protonation of amine groups of PHEG-Tyr nanogel and the expansion of PHEG-Tyr nanogel network due to predominant polymer–medium interactions [27]. The subsequent increase of pH to 7.4 led to the decrease of *D*_H_ of PHEG-Tyr nanogel to 131 nm driven by stronger polymer–polymer interactions including hydrophobic interactions and hydrogen bonds, leading to a shrinkage of the PHEG-Tyr nanogel [27]. The same trend was observed for the measurement of the pH-stimuli responsive behavior of *D*_H_ of the PHEG-Tyr nanogel at 37 °C (Figure 2a). Interestingly, DLS measurements revealed a thermal transition of PHEG-Tyr nanogel with significantly smaller *D*_H_, that is, 30–40 nm at 37 °C. This observation indicates that PHEG-Tyr nanogel is more solubilized at 25 °C than at 37 °C when the polymer–polymer interactions prevail [28]. The zeta potential of PHEG-Tyr nanogel was measured to determine the surface properties at different pH values and temperatures. Figure 2b shows that the zeta potential of PHEG-Tyr nanogel was not significantly affected by pH change. It was slightly anionic due to excess of carboxyl end groups and hydroxy groups [23]. Negligible difference could be seen between the measurements at 25 °C with an average zeta potential of ca. −7.5 mV and the measurements at 37 °C with an average zeta potential of ca. −9 mV. We suppose that PHEG-Tyr nanogel is more expanded due to protonation of the amine groups on side chains [23], which are closer to the surface of nanogel and lead to a slight increase of the zeta potential. When the pH increases, PHEG-Tyr nanogel shrinks and the amine groups on the side chains become more entangled inside the nanogel.

**Figure 3:**
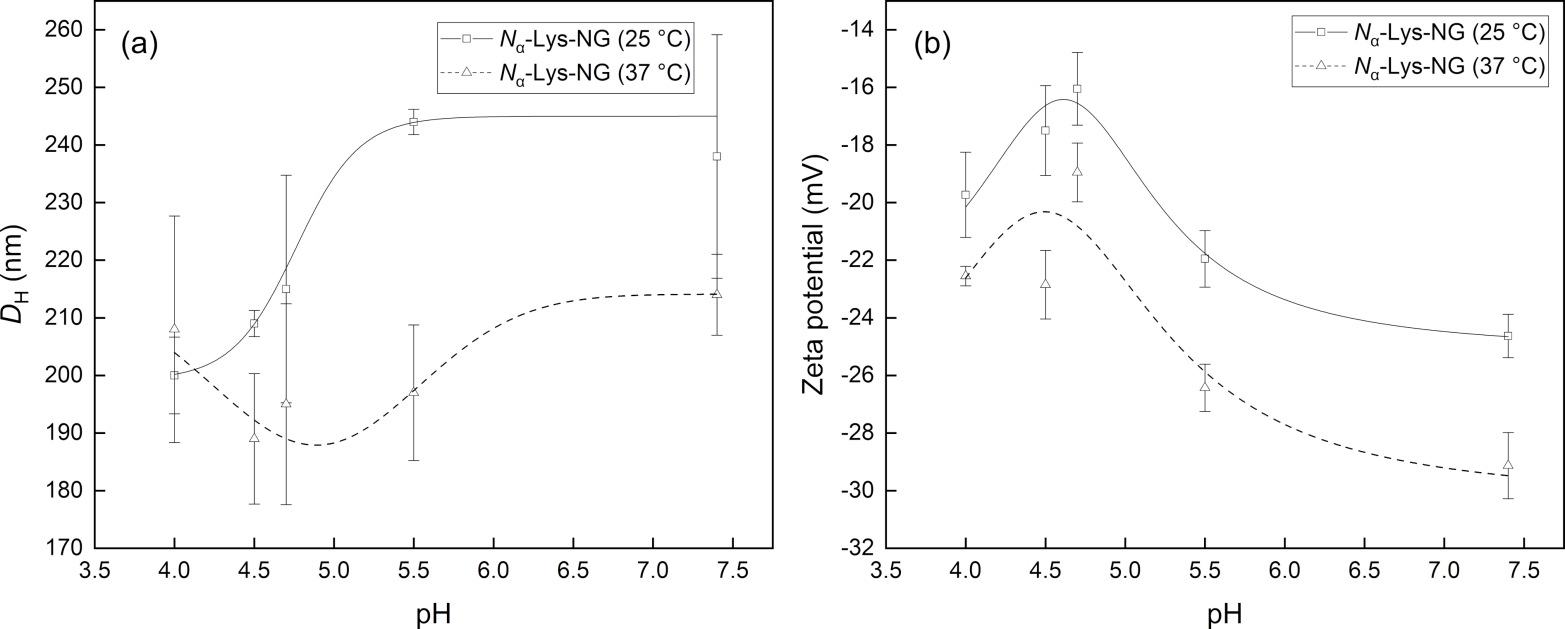
Dependence on the pH value of *D*_H_ (a) and zeta potential (b) of *N*^α^-Lys-NG nanogel at 25 (squares) and 37 °C (triangles). The lines are to guide the eye.

The measurement of the stimuli-responsive properties of *N*^α^-Lys-NG nanogel showed a different behavior compared to PHEG-Tyr nanogel (Figure 3a). An increase of *D*_H_ from 200 to 240 nm measured at 25 °C was observed when the pH value was raised from 4 to 7.4, as a result of prevailing polymer–medium interactions leading to a more expanded *N*^α^-Lys-NG nanogel structure. At 37 °C, the same trend of pH dependence of *D*_H_ was found and the diameter increased from 190 to 215 nm with elevated pH. The *D*_H_ values were also significantly smaller in comparison to the measurement at 25 °C as a result of the contribution from hydrophobic interactions and hydrogen bonds. However, it is important to note that the measurement was also affected by the broad particle size distribution of *N*^α^-Lys-NG nanogel documented by the error bars in Figure 3a. PDI values varied from 0.233 to 0.258 at 25 °C and from 0.233 to 0.289 at 37 °C within the tested pH range from 4 to 7.4. Therefore, we assumed that the dependence of *D*_H_ of *N*^α^-Lys-NG nanogel on the pH could be significantly distorted. The surface charge analysis showed that *N*^α^-Lys-NG nanogel was more anionic in comparison to PHEG-Tyr nanogel due to the presence of carboxyl groups of lysine in side chains of the zwitterionic *N*^α^-Lys-NG nanogel [25,29]. The zeta potential of *N*^α^-Lys-NG nanogel was not significantly affected by the change of temperature (Figure 3b). However, a slight decrease of zeta potential with increasing pH from 4 to 7.4, from approximately −17 to −25 mV, was observed, indicating a better colloidal stability of *N*^α^-Lys-NG nanogel at pH 7.4. This is caused by the fact that the polypeptide chains of *N*^α^-Lys-NG nanogel are more relaxed and expanded due to the presence of zwitterionic lysine-based substituents compared to PHEG-Tyr nanogel [25]. This assumption is also supported by the *D*_H_ dependence (Figure 3a) showing a *D*_H_ maximum at pH 7.4.

### Adsorption and release of BSA and AAT

**Figure 4:**
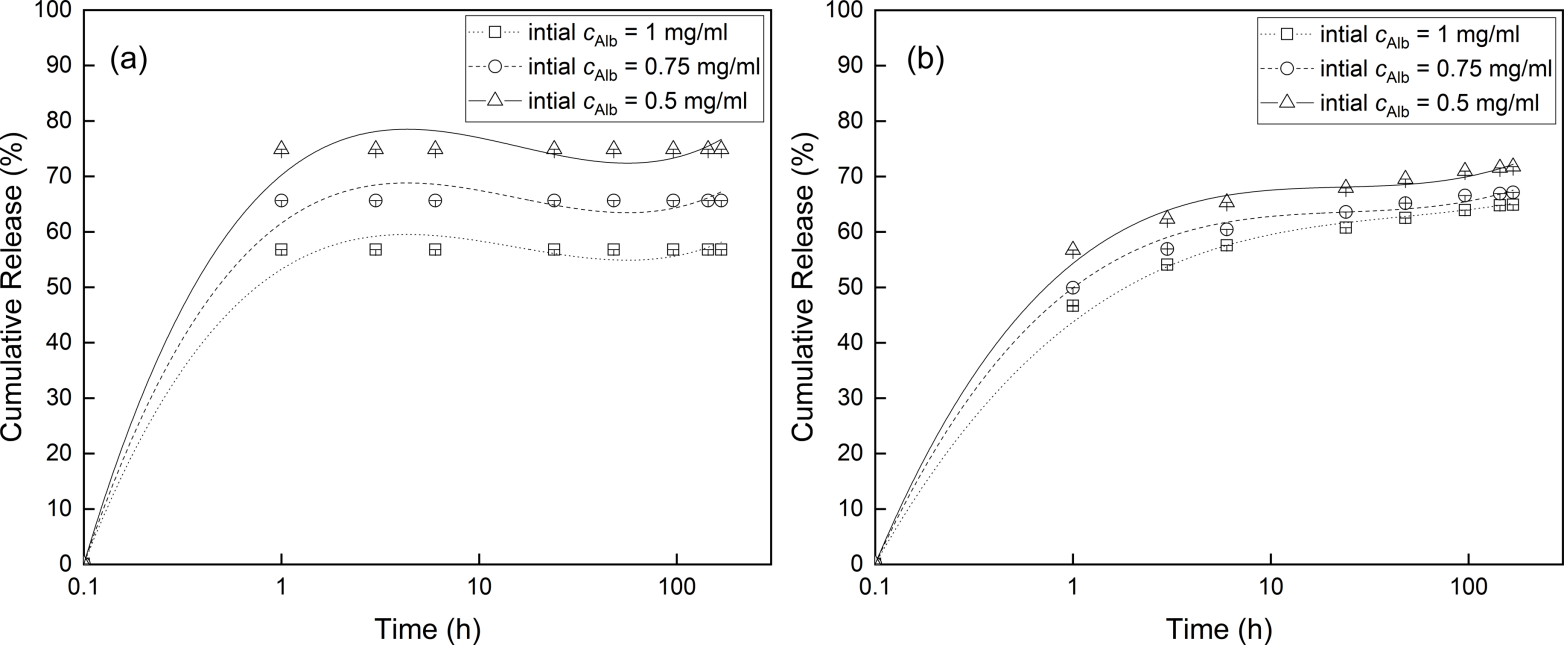
Release of ^125^I-radiolabeled albumin from PHEG-Tyr (a) and *N*^α^-Lys-NG (b) nanogels at initial ^125^I-radiolabeled albumin concentrations of 1 mg/mL (squares), 0.75 mg/mL (circles), and 0.5 mg/mL (triangles). The lines are to guide the eye.

Next, optimization studies of in vitro loading of ^125^I-radiolabeled BSA as a model protein and its release from PHEG-Tyr and *N*^α^-Lys-NG nanogels were performed. ^125^I-radiolabeled BSA was loaded onto PHEG-Tyr nanogel at pH 4.7 and 25 °C as PHEG-Tyr nanogel exhibits the most swollen state and a negative charge under these conditions (Figure 2). The uptake of BSA was found to be more efficient at or below the isoelectric point of BSA (pI 4.7) and, thus, BSA adsorption is driven by electrostatic and hydrophobic interactions [30–31]. PHEG-Tyr nanogel was incubated with three different ^125^I-radiolabeled BSA concentrations (1, 0.75, and 0.5 mg/mL), and the loading efficiency was found to be low, that is, 2.1% (21 µg/mL), 1.9% (15 µg/mL), and 3.3% (17 µg/mL), respectively. This could be explained by the weak interaction between ^125^I-radiolabeled BSA and PHEG-Tyr nanogel at pH 4.7 and 25 °C and the low capturing ability of PHEG-Tyr nanogel [32]. As a next step, PHEG-Tyr nanogel loaded with ^125^I-radiolabeled BSA was incubated at pH 7.4, simulating the physiological environment. A typical burst biphasic profile was observed with the burst release phase finishing within 6 h and followed by a power-law phase with constant release of ^125^I-radiolabeled BSA, after which equilibrium was reached (Figure 4a) [33]. The amount of released ^125^I-radiolabeled BSA increased with the decrease of initial concentration of ^125^I-radiolabeled BSA during loading onto the PHEG-Tyr nanogel. Furthermore, some amount of ^125^I-radiolabeled BSA was not released and remained entrapped in the PHEG-Tyr nanogel. This can be ascribed to the shrinkage of PHEG-Tyr nanogel after the change of pH from 4.7 to 7.4 (Figure 2a).

The next loading experiment showed that ^125^I-radiolabeled BSA has a greater affinity to the zwitterionic polyaspartamide nanogel *N*^α^-Lys-NG. In this case, loading of 1 and 0.75 mg/mL ^125^I-radiolabeled BSA resulted in average loading capacities of 11.1% (11.1 µg/mL) and 14.8% (11.1 µg/mL). As was observed in other works, a high loading efficiency of 35.9% (18 µg/mL) was found when *N*^α^-Lys-NG was incubated with 0.5 mg/mL ^125^I-radiolabeled BSA [31,34]. Interestingly, our results show that the amount of adsorbed ^125^I-radiolabeled BSA increased with the decrease of initial concentration of the ^125^I-radiolabeled BSA. The inverse effect was found when loading BSA onto poly(acrylic acid) and hybrid hydroxyapatite nanoparticles with chitosan/polyacrylic acid nanogels, where the loading was predominantly influenced by electrostatic interaction [35–36]. Our observation indicates that the adsorption of ^125^I-radiolabeled BSA onto *N*^α^-Lys-NG is mainly driven by hydrophobic interaction. At higher initial concentrations of ^125^I-radiolabeled BSA, both polypeptide nanogels, *N*^α^-Lys-NG and PHEG-Tyr, are quickly saturated and unable to adsorb higher amounts of ^125^I-radiolabeled BSA. A burst biphasic profile of adsorbed ^125^I-radiolabeled BSA was also observed, with the burst release phase occurring in the first 6 h (Figure 4b) followed by the power-law phase over the next 162 h with a total amount of ca. 65% released ^125^I-radiolabeled BSA. Clearly, some fraction of ^125^I-radiolabeled BSA was again entrapped in the shrunken *N*^α^-Lys-NG nanogel after the pH change (Figure 3a), probably due to high electrostatic interactions during the release at pH 7.4.

**Figure 5:**
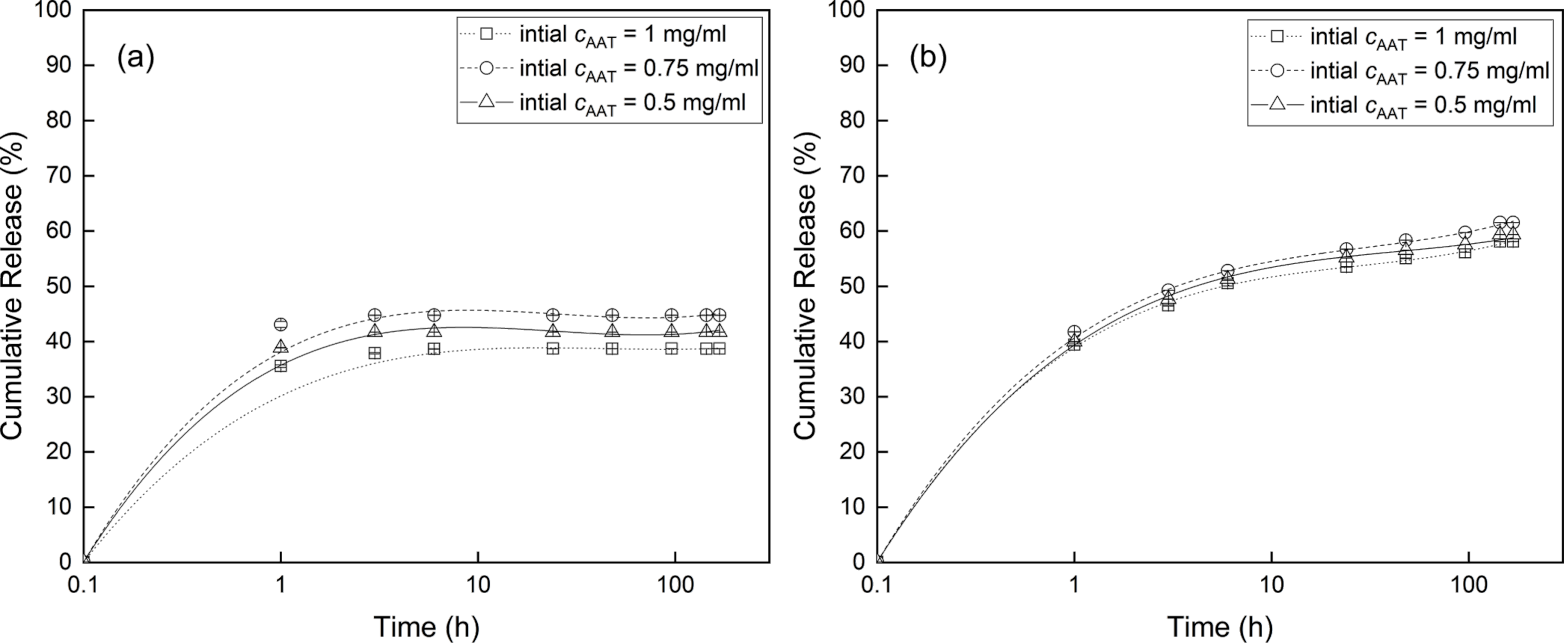
Release of ^125^I-radiolabeled α-1-antitrypsin from PHEG-Tyr (a) and *N*^α^-Lys-NG (b) nanogels at initial ^125^I-radiolabeled α-1-antitrypsin concentrations of 1 mg/mL (squares), 0.75 mg/mL (circles), and 0.5 mg/mL (triangles). The lines are to guide the eye.

After the loading and release procedure was optimized for BSA, PHEG-Tyr and *N*^α^-Lys-NG nanogels were incubated with three different concentrations of ^125^I-radiolabeled AAT (1, 0.75, and 0.5 mg/mL) to investigate their loading capacity and release profiles. Due to the fact that AAT has a pI in the range of 4.2–4.9, AAT was loaded at pH 4.7 as it was optimized with BSA to ensure efficient adsorption of AAT [31–32]. PHEG-Tyr nanogel again exhibited low adsorption of ^125^I-radiolabeled AAT (ca. 4 %) with 42, 23, and 22 µg/mL of loaded ^125^I-radiolabeled AAT, respectively. During the 168 h release study, the same release behavior was observed (Figure 5a) and approximately 35% of ^125^I-radiolabeled AAT was retained in the PHEG-Tyr nanogel after the release at pH 7.4. Presumably, the ^125^I-radiolabeled AAT was mainly adsorbed and released from the surface of the PHEG-Tyr nanogel while some fraction of the ^125^I-radiolabeled AAT was trapped in the collapsed PHEG-Tyr nanogel after the pH change (Figure 2a). The loading and release study showed the same trend, namely that the amount of released ^125^I-radiolabeled AAT increased with decreased initial concentration of ^125^I-radiolabeled AAT. Clearly, higher concentrations of ^125^I-labeled AAT lead to fast saturation of PHEG-Tyr due to immediate interactions that hinder subsequent adsorption of the ^125^I-radiolabeled AAT. In the case of *N*^α^-Lys-NG nanogel, the amount of adsorbed ^125^I-radiolabeled AAT increased from 11.9% (119 µg/mL) over 14.2% (106 µg/mL) to 20% (100 µg/mL) with the decrease of initial concentration of ^125^I-radiolabeled AAT (1, 0.75, and 0.5 mg/mL). Again, a burst biphasic release profile was observed. (Figure 5b). The release profile was not affected by the initial concentration of ^125^I-radiolabeled AAT. However, ca. 40% of ^125^I-radiolabeled AAT remained in the *N*^α^-Lys-NG nanogel after the release equilibrium was reached.

The loading and release study showed that zwitterionic polyaspartamide *N*^α^-Lys-NG nanogel exhibit a better loading capacity for ^125^I-radiolabeled BSA and ^125^I-radiolabeled AAT than PHEG-Tyr nanogel. Both polypeptide nanogels demonstrated burst biphasic release profiles, and our results were similar to the studies using PLGA particles as delivery system for AAT [21–22]. However, the PLGA particles did not exhibit any undesired entrapment of AAT as the polypeptide nanogels did. In contrast, our polypeptide nanogels are more advantageous due to the fact that they have a more favorable size, are soft, and demonstrate stimuli-responsive behavior. These properties and their release behavior of AAT makes them successful candidates for prompt inhibition of the inflammatory mediator trypsin.

### Pilot in vitro study of trypsin inhibition

**Figure 6:**
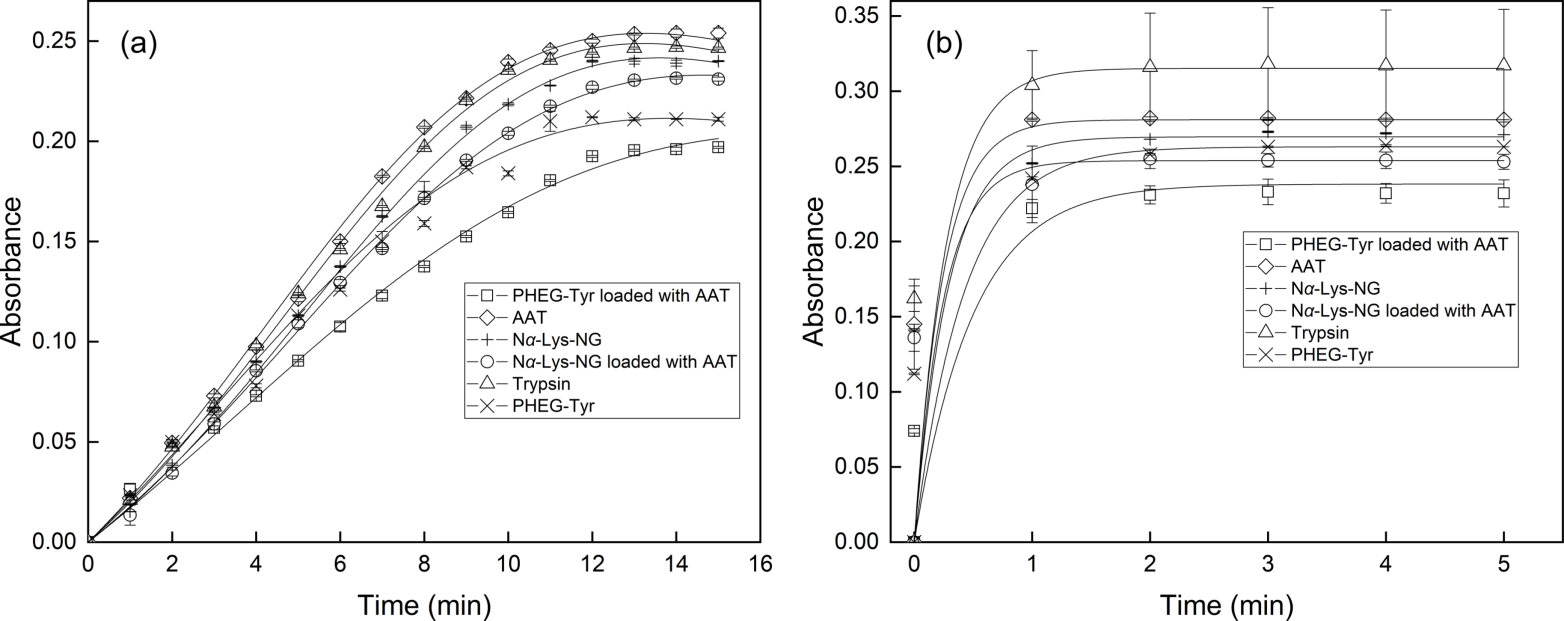
Spectrophotometric measurements of enzymatic assay of trypsin at trypsin/substrate ratio 1:50 (a) and 1:25 (b) using trypsin without inhibition (triangles), AAT (diamonds), *N*^α^-Lys-NG nanogel loaded with AAT (circles), PHEG-Tyr nanogel loaded with AAT (squares), *N*^α^-Lys-NG nanogel (plus signs), and PHEG-Tyr nanogel (crosses). The lines are to guide the eye.

As a next step, the inhibition activity of PHEG-Tyr and *N*^α^-Lys-NG nanogels loaded with AAT was investigated by spectrophotometric measurement at λ = 253 nm according to the modified procedure of enzymatic assay of trypsin. The trypsin inhibition study was carried out at two trypsin/substrate ratios of 1:50 and 1:25. The trypsin/AAT ratio was 1:1 for both trypsin/substrate ratios. According to the previous loading studies of the ^125^I-radiolabeled ATT onto PHEG-Tyr and *N*^α^-Lys-NG nanogels, the maximum loading capacities of AAT were found, and the corresponding amounts of PHEG-Tyr and *N*^α^-Lys-NG nanogels were calculated. In each pilot trypsin inhibition assay, PHEG-Tyr nanogel loaded with AAT (Figure 6, squares), *N*^α^-Lys-NG nanogel loaded with AAT (Figure 6, circles), PHEG-Tyr nanogel (Figure 6, crosses), *N*^α^-Lys-NG nanogel (Figure 6, plus signs), AAT (Figure 6, diamonds), and conversion of BAEE substrate with trypsin without inhibition (Figure 6, triangles) were tested.

At a trypsin/substrate ratio of 1:50, the maximum conversion of BAEE with trypsin without inhibition was reached after 15 min (Figure 6a, triangles). When only AAT was added, the consumption of BAEE by trypsin was not affected due to the low concentration of the inhibitor (Figure 6a, diamonds). We observed that the trypsin activity was slightly inhibited by *N*^α^-Lys-NG loaded with AAT (Figure 6a, circles), and the inhibition effect was more pronounced when PHEG-Tyr nanogel loaded with AAT was used (Figure 6a, curve squares). Then, the inhibition effect of both non-loaded nanogels, *N*^α^-Lys-NG (Figure 6a, plus signs) and PHEG-Tyr (Figure 6a, crosses) was tested. Interestingly, it was found that these polypeptide nanogels also showed an inhibition effect on trypsin. The results indicate that these nanogels synthesized from *N*^α^-Lys-NG or PHEG-Tyr polypeptide precursors, in other words from synthetic poly(amino acid)s, have the ability to inhibit the trypsin active site because they contain positively charged amino acids (containing NH_2_ terminal groups) and aromatic amino acids [23,25]. It is known that inhibitors containing these amino acids in their structure can block the active site of trypsin because they inhibit the substrate receptor at the trypsin active site [37]. According to our observations, the inhibition effect was more profound when AAT was combined with each nanogel.

For a trypsin/substrate ratio 1:25, full conversion of BAEE with trypsin without inhibition was achieved within 3 min (Figure 6b, triangles). This trypsin/substrate ratio was found to be the most favorable for further inhibition studies. Clearly, AAT decreased the activity of trypsin only slightly (Figure 6b, diamonds), while increased inhibition was observed when AAT was loaded onto both tested nanogels. PHEG-Tyr nanogel (Figure 6a, squares) demonstrated higher inhibition activity than *N*^α^-Lys-NG nanogel (Figure 6b, circles). Interestingly, this observation proves that the inhibition activity of ATT is reinforced by *N*^α^-Lys-NG and even more by PHEG-Tyr, probably because their negative charge facilitates positioning of ATT and blockage of the trypsin active site [8]. The inhibition effect of both non-loaded nanogels, *N*^α^-Lys-NG (Figure 6b, plus signs) and PHEG-Tyr (Figure 6b, crosses) was tested again confirming the inhibition activity of these polypeptide nanogels. These results prove that the developed nanogel systems loaded with AAT can even more effectively block the active site of trypsin and inhibit its enzymatic activity during conversion of BAEE substrate.

We deduced that the inhibition activity of the developed PHEG-Tyr nanogel system with AAT is ensured by the combination of suitable properties of PHEG-Tyr nanogel itself and AAT loaded onto PHEG-Tyr nanogel. This pilot in vitro study was crucial for the development of functional, fully biocompatible, and biodegradable nanogel carriers. The study will serve for subsequent optimization of the inhibition effect of PHEG-Tyr nanogel loaded with AAT at varying trypsin/substrate ratios using different amounts of PHEG-Tyr and AAT and, eventually, in vivo inhibition studies with animal models.

## Conclusion

In this study, newly developed hydrophilic, biocompatible polyaspartamide *N*^α^-Lys-NG, and hydrophilic, biocompatible, and biodegradable PHEG-Tyr nanogel delivery systems for AAT were prepared by HRP/H_2_O_2_-mediated crosslinking in inverse miniemulsion. Both negatively charged polypeptide nanogels demonstrated pH stimuli-responsive behavior and thermal transitions, verified by changed hydrodynamic diameters. Loading and release were first optimized with BSA, after which loading and release of AAT were studied. The results show that AAT has an affinity to both nanogels. A burst release of ATT in the first 6 h, followed by continuous release over ca. 160 h, was observed. Finally, both nanogels were used as AAT depots for the inhibition of the inflammatory mediator trypsin. The pilot in vitro enzymatic study proved the ability of the nanogels loaded with AAT to successfully inhibit trypsin and showed that both AAT-loaded nanogels have better inhibition activity than sole AAT. Moreover, this pilot study revealed that both non-loaded polypeptide nanogels also inhibit the enzymatic activity of trypsin. The observed properties of this delivery system will be further studied to better understand the mechanism of inhibition action. This fully biocompatible nanogel system might be a potential candidate for the development of new systems inhibiting the inflammatory mediator trypsin and, possibly, for the treatment of pancreatitis. Further studies will serve for future optimization of the new nanogel systems. The optimum ratio between trypsin and AAT-loaded nanogels and the inhibition activity of the delivery system in the pH range from 7 and 7.2, which is also relevant for the development of pancreatitis, will be determined. Finally, in vivo experiments with animal models will be conducted.

## Experimental

### Materials

Benzene p.a., cyclohexane p.a. (CHX), chloroform p.a., *N,N*-dimethylformamide p.a. (DMF), 1,4-dioxane p.a., methanol p.a., and tetrahydrofuran p.a. (THF) were obtained from Lach-Ner (Czech Republic). The solvents were purified and dried by a standard procedure before use. Aminopropan-2-ol (purified by vacuum distillation) was purchased from Alfa Aesar (Kandel, Germany). 1,6-Diaminohexane, HBr 33 wt % in acetic acid, 30% hydrogen peroxide solution (w/w) in water containing stabilizer (H_2_O_2_), horseradish peroxidase type VI (HRP), α_1_-antitrypsin from human plasma (AAT), trypsin from bovine pancreas type I, propargylamine, ethanolamine, *N*^α^-benzoyl-ʟ-arginine ethyl ester hydrochloride (BAEE), *N*^α^-(*tert*-butoxycarbonyl)-ʟ-lysine (Boc-Lys-OH), polyoxyethylenesorbitan trioleate (TWEEN 85), sodium methoxide, sorbitan monooleate (SPAN 80), triphosgene, and tyramine were purchased from Sigma-Aldrich (Prague, Czech Republic) and were used without purification. γ-Benzyl-ʟ-glutamate (BLG) 99% was purchased from Emmenar Bio-Tech (Sanathnagar, India) and was recrystallized from a hot water/ethanol mixture. OxymaPure was purchased from Irish Biotech (Marktredwitz, Germany). PD-10 desalting columns (Sephadex G 25) were obtained from Amersham Biosciences (Uppsala, Sweden). Na^125^I-radiolabeling solution (3 700 MBq/mL) was purchased from the Institute of Isotopes (Budapest, Hungary). Pierce™ Iodination Beads were received from Thermo Fisher Scientific.

### Preparation of nanogels

The PHEG-Tyr polymer precursor containing 10.8 wt % of Tyr units was prepared and characterized according to a previously published procedure [23]. PHEG-Tyr nanogel was prepared by HRP/H_2_O_2_-mediated crosslinking in inverse miniemulsion by modification of an earlier procedure [24]. Briefly, PHEG-Tyr (0.18 g) was dissolved in Q-H_2_O (4 g) in a round-bottom glass flask for 24 h at room temperature under magnetic stirring. Then, HRP (0.450 mg) was dissolved in Q-H_2_O (0.32 g) and added to the PHEG-Tyr aqueous solution. The surfactants SPAN 80 (0.855 g) and TWEEN 85 (0.045 g) were dissolved in CHX (20.4 g) and mixed with the aqueous solution of PHEG-Tyr with HRP. This two-phase system was cooled to 0 °C and dispersed using a UP200Ht ultrasonic processor equipped with a sonotrode S26d7 (Hielscher Ultrasonics GmbH, Teltow, Germany) for 150 s at an amplitude of 40%. The formed inverse miniemulsion was transferred into a 30 mL glass reaction vessel equipped with an anchor-type stirrer (500 rpm). Finally, H_2_O_2_ (16 µL) was added with a syringe, and the nanogelation was carried out at room temperature for 2 h. The nanogel was separated by centrifugation (11,000 rpm/20 min), washed three times with CHX, dispersed in CHX (20 mL) overnight, and again washed seven times with CHX to completely remove any residual surfactant. Then, the nanogel was dispersed in distilled water and dialyzed (molecular weight cut-off: <100 kDa) against distilled water for seven days. Finally, the nanogel was freeze-dried from distilled water.

The zwitterionic polyaspartamide nanogel (*N*^α^-Lys-NG) was prepared by the indirect strategy from polyaspartamide with protected zwitterionic groups (*N*^α^-Lys-P-HE-TyrAA) with the following deprotection step leading to the formation of nanogel with zwitterionic groups according to our earlier published procedure [25].

### Characterization of nanogels

DLS and zeta potential measurements were performed with dispersions of PHEG-Tyr and *N*^α-^Lys-NG nanogels in PBS buffer at pH 4, 4.5, 4.7, 5.5, and 7.4 (1 mg/mL) at 25 and 37 °C using a Zetasizer Nano ZS (Malvern Instruments Ltd., Worcestershire, UK) at 633 nm and 173° detection angle. Size and size distribution were obtained from the correlation function using CONTIN analysis available in the Malvern software. The hydrodynamic diameters (*D*_H_) were calculated using the Stokes–Einstein equation. For DLS measurements, both nanogels were dispersed in Q-H_2_O (2 mL; 1 mg/mL) in glass vials by a UP200Ht ultrasonic processor equipped with a sonotrode Hielscher S26d2 for 5 min at 40% amplitude using the following method: 2 min pulsation (0.5 s pulse rate), 2 min without pulsation, and 1 min pulsation (0.5 s pulse rate). All measurements were performed in pentaplicates, and data were expressed as mean and standard deviation.

Morphology, size, and particle size distribution of the nanogels were studied using a Tecnai G2 Spirit Twin 12 transmission electron microscope (TEM; FEI; Brno, Czech Republic) after negative staining of nanogel samples with uranyl acetate. The number-average diameter (*D*_n_), weight-average diameter (*D*_w_), and dispersity (*Ð*) were calculated using ImageJ software by counting the hydrogel nanoparticles in the TEM images following these equations:


[1] $$D_{n}=\frac{\sum n_{i}D_{i}}{\sum n_{i}},$$

[2] $$D_{\text{w}}=\frac{\sum n_{i}D_{i}^{4}}{\sum n_{i}D_{i}^{3}},$$

[3] $$Ð=\frac{D_{\text{w}}}{D_{n}},$$

where *n*_i_ and *D*_i_ are the number and diameter of the *i*-th microsphere, respectively.

### ^125^I-radiolabeling and loading and release of BSA and AAT

A solution of BSA (10 mg), or AAT (10 mg), in PBS buffer (400 µL, pH 7.4) was reacted with [^125^I]-NaI solution (155 MBq) for 30 min in the presence of two IODO-BEADS (pre-washed with PBS buffer, pH 7.4). After the separation of the IODO-BEADS, a solution of ascorbic acid (10 µL, 25 mg/mL in PBS buffer, pH 7.4) was added to the ^125^I-radiolabeled BSA, or ^125^I-radiolabeled AAT, and incubated for 30 min. Finally, the ^125^I-radiolabeled BSA, or ^125^I-radiolabeled AAT, was separated with a PD10 desalting column to remove impurities, unreacted compounds, and low molecular fractions. The purified fraction of the ^125^I-radiolabeled BSA, or ^125^I-radiolabeled AAT, was used in the following experiments.

PHEG-Tyr nanogel dispersion (0.5 mL, 3 mg/mL in PBS buffer with pH 4.7) was mixed with solutions of ^125^I-radiolabeled BSA (0.5 mL), or ^125^I-radiolabeled AAT (0.5 mL), in PBS buffer (pH 7.4) to obtain solutions with three different BSA concentrations (1, 0.75, and 0.5 mg/mL) in separate microtubes. The pH value of the loading assays was adjusted to pH 4.7 with the addition of 5 µL of 1 M HCl. The final concentration of PHEG-Tyr nanogel in the loading assays was 1.5 mg/mL. The loading of ^125^I-radiolabeled BSA, or ^125^I-radiolabeled AAT, was performed for 24 h at 25 °C under mild shaking. Then, the loading assays were centrifuged (7,000 rpm/10 min). Supernatants were removed with a micropipette and placed in clear microtubes. The radioactivity of PHEG-Tyr nanogels and supernatants was measured using a 2480 Wizard2^®^ Automatic Gamma Counter (PerkinElmer, Massachusetts, USA) to determine the loading capacity of PHEG-Tyr nanogel.

PHEG-Tyr nanogels loaded with ^125^I-radiolabeled BSA, or ^125^I-radiolabeled AAT, were re-dispersed in fresh PBS buffer (0.5 mL) to study the release profile at 25 °C. At exact time intervals (*t* = 1, 3, 6, 24, 48, 96, 144, and 168 h), the PHEG-Tyr nanogel was removed by centrifugation (7,000 rpm/10 min) and the radioactivity of the supernatant with released ^125^I-radiolabeled BSA, or ^125^I-radiolabeled AAT, and the radioactivity of the PHEG-Tyr nanogel pellet loaded with ^125^I-radiolabeled BSA, or ^125^I-radiolabeled AAT, were measured using a 2480 Wizard2^®^ Automatic Gamma Counter. Then, the PHEG-Tyr nanogel was re-dispersed in fresh PBS buffer (0.5 ml, pH 7.4) to continue the release study.

Loading of ^125^I-radiolabeled BSA, or ^125^I-radiolabeled AAT using *N*^α^-Lys-NG nanogel was performed according to the same procedure.

All measurements were performed in triplicates and data were expressed as mean and standard deviation.

### Spectrophotometric measurements of inhibition of trypsin enzymatic activity

Inhibition assays were carried out at trypsin/substrate molar ratios of 1:50 and 1:25. The trypsin/AAT molar ratio was 1:1. First, PHEG-Tyr (6.97 mg and 3.48 mg) or *N*^α^-Lys-NG (1.53 mg and 0.77 mg) were dispersed in PBS (4.5 mL, pH 4.7) and loaded with AAT (0.102 and 0.051 mg) for 24 h. The assay was prepared by mixing of BAEE solution (2 mL, 0.37 mM) in PBS buffer (pH 7.6) with 1 mM HCl (0.125 mL), dispersion of AAT loaded-PHEG-Tyr, or AAT loaded-*N*^α^-Lys-NG, nanogel (1 mL), and trypsin solution (0.075 mL) in 1 mM HCl in cuvettes for UV–vis spectrophotometric measurements. Immediately after mixing, the absorbance at λ = 253 nm was recorded for 5 or 15 min using 1 min time intervals at 25 °C. A blank solution was prepared by mixing *N*^α^-benzoyl-ʟ-arginine ethyl ester substrate solution (2 mL, 0.375 mM) in PBS buffer (pH 7.6) with 1 mM HCl (0.125 mL), and dispersion of AAT loaded-PHEG-Tyr, or AAT loaded-*N*^α-^Lys-NG, nanogel (1 mL). Inhibition assays using only AAT without the nanogels were prepared by solving AAT (0.102 and 0.051 mg) in PBS buffer (4 mL, pH 4.7) following the same procedure. Trypsin enzymatic assay was prepared by mixing BAEE solution (3 mL, 0.375 mM) in PBS buffer (pH 7.6) with 1 mM HCl (0.125 mL) and trypsin solution (0.075 mL) in 1 mM HCl in cuvettes for UV–vis spectrophotometric measurements. Immediately after mixing, the absorbance at *λ* = 253 nm was recorded for 5 or 15 min using 1 min time intervals. The blank solutions were prepared without the addition of trypsin and with 0.2 mL 1 mM HCl. All measurements were performed in triplicates and data were expressed as mean and standard deviation.

## References

[1] Ouyang G, Pan G, Liu Q, Wu Y, Liu Z, Lu W, Li S, Zhou Z, Wen Y (2020). BMC Med.

[2] Drake M, Dodwad S-J M, Davis J, Kao L S, Cao Y, Ko T C (2021). J Clin Med.

[3] Xiao A Y, Tan M L Y, Wu L M, Asrani V M, Windsor J A, Yadav D, Petrov M S (2016). Lancet Gastroenterol Hepatol.

[4] Szabó A, Toldi V, Gazda L D, Demcsák A, Tőzsér J, Sahin-Tóth M (2021). J Biol Chem.

[5] Gui F, Zhang Y, Wan J, Zhan X, Yao Y, Li Y, Haddock A N, Shi J, Guo J, Chen J (2020). J Clin Invest.

[6] Brandl T, Simic O, Skaanderup P R, Namoto K, Berst F, Ehrhardt C, Schiering N, Mueller I, Woelcke J (2016). Bioorg Med Chem Lett.

[7] Liu K (2021). J Am Oil Chem Soc.

[8] Pouvreau L, Chobert J-M, Briand L, Quillien L, Tran V, Guéguen J, Haertlé T (1998). FEBS Lett.

[9] Chanphai P, Tajmir-Riahi H A (2016). Carbohydr Polym.

[10] Mao X, Yang Z (2021). Ann Palliat Med.

[11] Beghdadi W, Madjene L C, Benhamou M, Charles N, Gautier N, Launay P, Blank U (2011). Front Immunol.

[12] Hashimoto Y, Mukai S-a, Sasaki Y, Akiyoshi K (2018). Adv Healthcare Mater.

[13] Vashist A, Kaushik A, Vashist A, Bala J, Nikkhah-Moshaie R, Sagar V, Nair M (2018). Drug Discovery Today.

[14] Massi L, Najer A, Chapman R, Spicer C D, Nele V, Che J, Booth M A, Doutch J J, Stevens M M (2020). J Mater Chem B.

[15] Ozawa Y, Sawada S-i, Morimoto N, Akiyoshi K (2009). Macromol Biosci.

[16] Hirakura T, Yasugi K, Nemoto T, Sato M, Shimoboji T, Aso Y, Morimoto N, Akiyoshi K (2010). J Controlled Release.

[17] Morimoto N, Hirano S, Takahashi H, Loethen S, Thompson D H, Akiyoshi K (2013). Biomacromolecules.

[18] Alkanawati M S, Machtakova M, Landfester K, Thérien-Aubin H (2021). Biomacromolecules.

[19] Dunlea D M, Fee L T, McEnery T, McElvaney N G, Reeves E P (2018). J Inflammation Res.

[20] Stockley R A (2015). Ann Transl Med.

[21] Pirooznia N, Hasannia S, Lotfi A S, Ghanei M (2012). J Nanobiotechnol.

[22] Arjmand S, Bidram E, Lotfi A S, Mahdavi H, Alavi M (2011). Int J Biosci, Biochem Bioinf.

[23] Dvořáková J, Šálek P, Korecká L, Pavlova E, Černoch P, Janoušková O, Koutníková B, Proks V (2020). J Appl Polym Sci.

[24] Oleshchuk D, Šálek P, Dvořáková J, Kučka J, Pavlova E, Francová P, Šefc L, Proks V (2021). Mater Sci Eng, C.

[25] Hladysh S, Oleshchuk D, Dvořáková J, Golunova A, Šálek P, Pánek J, Janoušková O, Kaňková D, Pavlova E, Proks V (2021). Eur Polym J.

[26] Marciel A B, Chung E J, Brettmann B K, Leon L (2017). Adv Colloid Interface Sci.

[27] Arteche Pujana M, Pérez-Álvarez L, Cesteros Iturbe L C, Katime I (2014). Eur Polym J.

[28] Bordat A, Boissenot T, Nicolas J, Tsapis N (2019). Adv Drug Delivery Rev.

[29] Ostolska I, Wiśniewska M (2014). Colloid Polym Sci.

[30] Argentiere S, Blasi L, Ciccarella G, Barbarella G, Cingolani R, Gigli G (2010). J Appl Polym Sci.

[31] Zhang Y, Zhang D, Wang J-T, Zhang X, Yang Y (2021). Polym Chem.

[32] Wang Q, Xu H, Yang X, Yang Y (2008). Int J Pharm.

[33] Yoo J, Won Y-Y (2020). ACS Biomater Sci Eng.

[34] Lale S V, Koul V, Thakur V K, Thakur M K, Voicu S I (2018). Stimuli-Responsive Polymeric Nanoparticles for Cancer Therapy. Polymer Gels, Prospectives and Applications.

[35] Chen Y, Zheng X, Qian H, Mao Z, Ding D, Jiang X (2010). ACS Appl Mater Interfaces.

[36] Qin J, Zhong Z, Ma J (2016). Mater Sci Eng, C.

[37] Cohen A B (1973). J Biol Chem.

